# Polyketide stereocontrol: a study in chemical biology

**DOI:** 10.3762/bjoc.13.39

**Published:** 2017-02-24

**Authors:** Kira J Weissman

**Affiliations:** 1 UMR 7365, Ingénierie Moléculaire et Physiopathologie Articulaire (IMoPA), CNRS-Université de Lorraine, Biopôle de l’Université de Lorraine, Campus Biologie Santé, Avenue de la Forêt de Haye, BP 50184, 54505 Vandœuvre-lès-Nancy Cedex, France

**Keywords:** chemical biology, polyketide synthases, reduced polyketides, stereocontrol

## Abstract

The biosynthesis of reduced polyketides in bacteria by modular polyketide synthases (PKSs) proceeds with exquisite stereocontrol. As the stereochemistry is intimately linked to the strong bioactivity of these molecules, the origins of stereochemical control are of significant interest in attempts to create derivatives of these compounds by genetic engineering. In this review, we discuss the current state of knowledge regarding this key aspect of the biosynthetic pathways. Given that much of this information has been obtained using chemical biology tools, work in this area serves as a showcase for the power of this approach to provide answers to fundamental biological questions.

## Introduction

**Figure 1:**
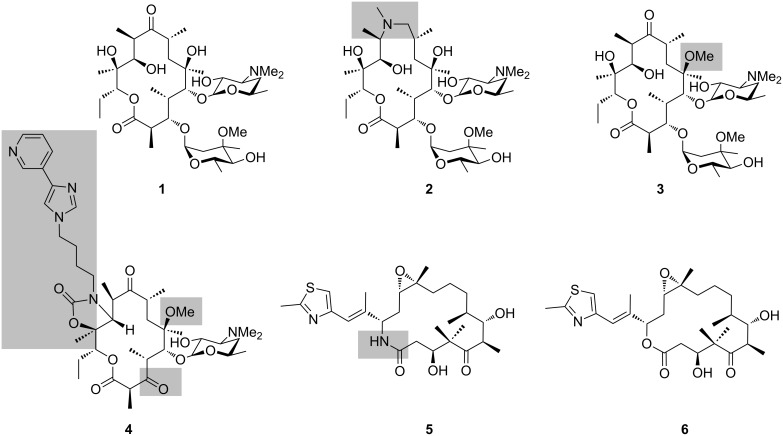
Structures of clinically-relevant polyketides: erythromycin A (**1**), azithromycin (**2**), clarithromycin (**3**), telithromycin (**4**), and ixabepilone (**5**) (a semi-synthetic derivative of epothilone B (**6**)). The structural variations relative to the parent compounds **1** and **6** are indicated by the grey boxes.

Reduced polyketides and their derivatives form the basis for a number of medicines in current clinical usage, notably anti-infectives [1] (e.g., erythromycin A (**1**) and its semi-synthetic derivatives azithromycin (**2**), clarithromycin (**3**), telithromycin (**4**) ([2] and others) and anticancer compounds (e.g., ixabepilone (**5**) [3]), a semi-synthetic derivative of the natural product epothilone B (**6**)) (Figure 1). Given the medical and economic importance of these compounds, there is significant interest in trying to generate new versions of polyketides for evaluation as drug leads. The significant bioactivity of these compounds derives from their complex structures (particularly when compared to the typical products of chemical synthesis [4]), which incorporate both high functional group density and rich stereochemistry. These features, coupled with the fact that the majority of reduced polyketides are macrocyclic, result in significant in-built conformational constraints. As a consequence, these molecules present their diverse functionality in a defined way in three dimensions, allowing them to bind their biological targets with useful affinity (10^−7^ to 10^−9^ M [4]).

**Figure 2:**
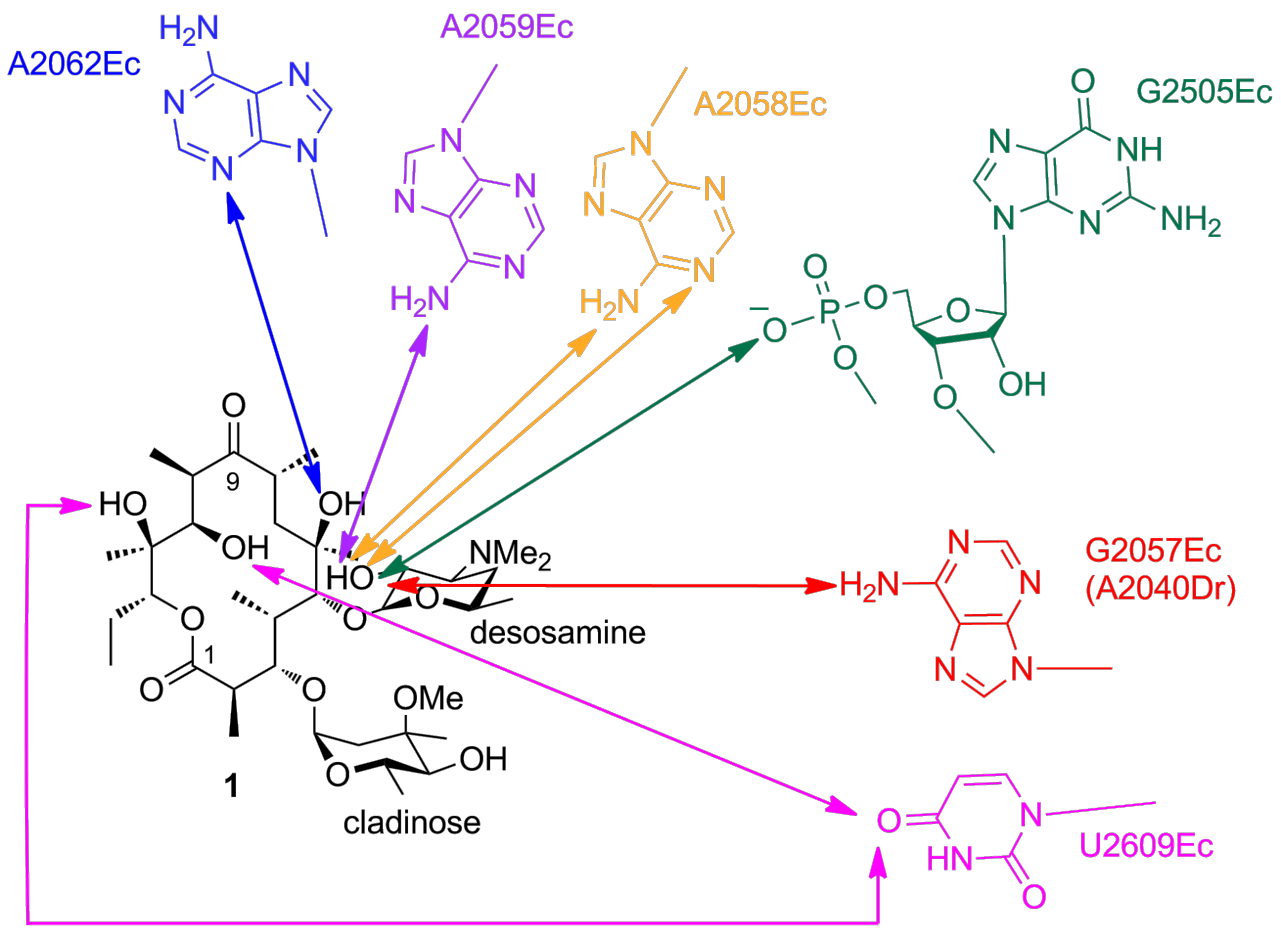
Schematic of erythromycin A (**1**) bound to 23S ribosomal RNA of the 50S subunit of the *Deinococcus radiodurans* (Dr) ribosome. The interactions between the polyketide and the nucleotides (*Escherichia coli* (Ec) numbering) are indicated with colored arrows (reactive groups are less than 4.4 Å apart). Adapted from [5].

Erythromycin A (**1**, Figure 1) is the prototypical polyketide, as its biosynthesis has been studied most heavily to date. The structure incorporates 10 stereocenters, and so in principle, 1024 (2^10^) different stereoisomers are possible. Yet, nature reliably assembles only one stereoisomer (at least at detectable levels), at once revealing the strict stereocontrol underpinning the pathway and the importance of synthesizing this particular version. Indeed, the crystal structure of erythromycin A (**1**) bound to the 50S ribosomal subunit of the eubacterium *Deinococcus radiodurans* [5], shows a suite of interactions between the ribosomal bases and multiple chiral functional groups of the polyketide macrolactone, including the hydroxy groups at C-6, C-11 and C-12, and the desosamine appended to the hydroxy group at C-5 (whose positioning in 3D depends on the hydroxy stereochemistry) (Figure 2).

This intimate link between polyketide stereochemistry and biological activity makes the control of stereochemistry an attractive research area for attempts to generate new polyketide structures by synthetic biology [6]. The aim of this review is to trace how our understanding of these feature of the biosynthesis has developed, and more specifically, the critical role that an array of chemical biology approaches [7] has played in furnishing the underlying data. These include, but are not limited to, the synthesis of isotopically-labeled precursors and the analysis of the resulting labeling patterns, characterization by assays in vitro of wild type and mutant recombinant enzymes in the presence of synthetic substrates, and genetic engineering of model systems coupled with analysis of product structures by gas-chromatography/mass spectrometry (GC–MS) and liquid chromatography (LC)–MS.

## Review

### Biosynthesis of complex polyketides by modular PKSs and stereochemical considerations

The reduced or complex class of polyketides is assembled in bacteria by gigantic multienzymes called polyketide synthases (PKSs), in a process resembling fatty acid biosynthesis by the mammalian fatty acid synthase (FAS) [8] from which the PKSs likely evolved [9]. In both cases, simple acyl-CoA building blocks are concatenated head-to-tail to construct linear chains. Several features distinguish these two pathways, however: PKSs use a wider range of both initial building blocks (referred to as ‘starter units’) and chain extension units than their FAS counterparts which notably results in branching of the chains, the degree of reduction of the initially formed C3-keto intermediates is variable (whereas in FA biosynthesis, full reduction to the fatty acyl group occurs systematically), and polyketides are most typically released in cyclic form, whereas fatty acids are liberated as carboxylic acids. The much more complicated biosynthetic control in PKSs is achieved by successive action of multiple FAS-like modules (hence the name ‘modular PKS’ for this type of system), each of which carries out a single round of chain extension and chemical tailoring of the resulting intermediate.

**Figure 3:**
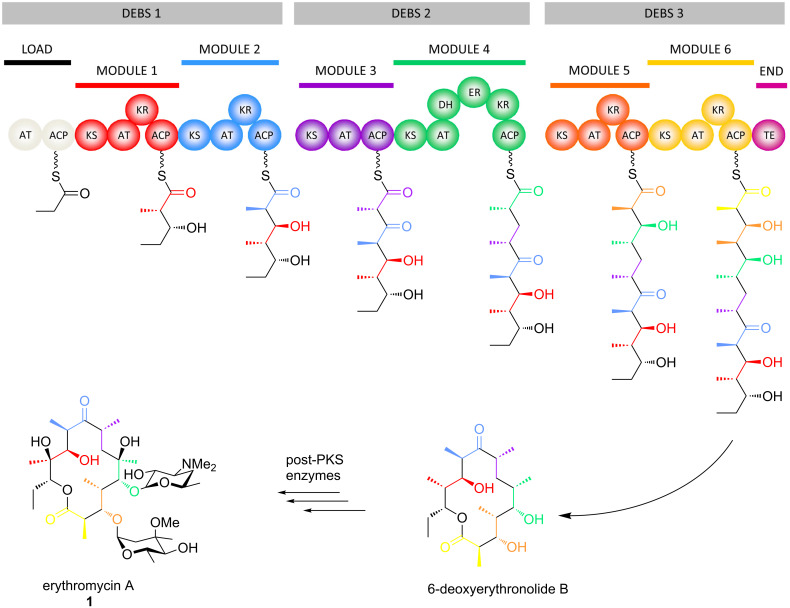
Schematic of the biosynthetic pathway leading to erythromycin A (**1**) in the bacterium *Saccharopolyspora erythraea*. The first stage of biosynthesis occurs on a modular polyketide synthase (PKS) incorporating three gigantic multienzyme polypeptides, DEBS 1, 2 and 3. Each of these subunits contains two chain extension modules, while DEBS 1 additionally incorporates a loading module to initiate the biosynthesis, and DEBS 3, a termination module, consisting of a thioesterase (TE) domain. Each of the chain extension modules includes three essential domains (ketosynthase (KS), acyl transferase (AT) and acyl carrier protein (ACP)), and a variable complement of processing activities (ketoreductase (KR), dehydratase (DH) and enoyl reductase (ER)). The number and character of the variable domains correlates precisely with the structure of the resulting intermediates (the building block added at each stage is color-coded to match the module responsible). Following release of the chain from the PKS, the first free intermediate, 6-deoxyerythronolide B, is further modified by a series of post-PKS enzymes to yield the final, bioactive metabolite.

Each PKS module incorporates three functional domains necessary for chain growth (Figure 3): an acyl transferase (AT) which selects the appropriate precursor from the cellular pool, a ketosynthase (KS) which extends the chain via a Claisen-like decarboxylative condensation, and a non-catalytic acyl carrier protein (ACP) to which the intermediates are covalently tethered through a phosphopantetheine prosthetic group. The modules can also incorporate a variable complement of the processing activities which act in each cycle of FA biosynthesis, including ketoreductase (KR), dehydratase (DH) and enoyl reductase (ER) domains; these activities lead successively to hydroxy groups, olefinic moieties or saturated methylene groups at specific positions in the polyketide chains. Building of the polyketide core is typically terminated by a thioesterase (TE) domain situated at the end of the final PKS multienzyme, which releases the product by hydrolysis or more usually macrolactonization, using an internal hydroxy nucleophile. This PKS-free intermediate (6-deoxyerythronolide B in the case of erythromycin biosynthesis, Figure 3) is then frequently modified by a series of so-called ‘post-PKS enzymes’ (e.g., methyl transferases, hydroxylases, and glycosyl transferases), to achieve its final bioactive form [10].

**Figure 4:**
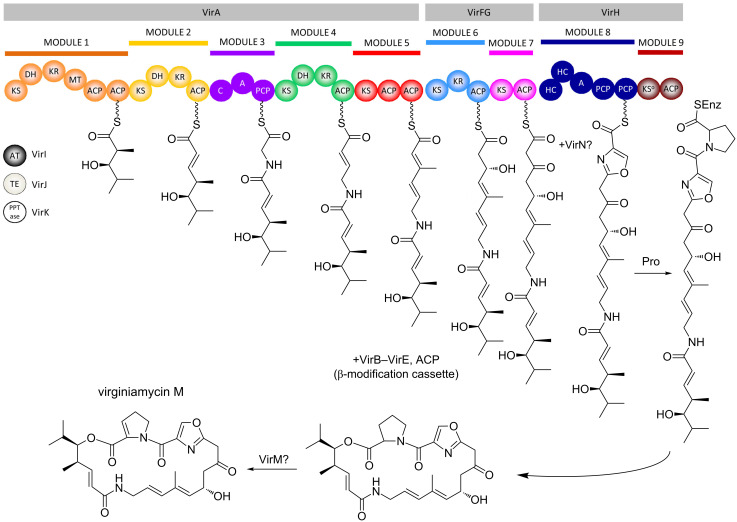
Schematic of the virginiamycin PKS from *Streptomyces virginiae*, a member of the *trans*-AT PKS family [11]. The PKS comprises at least three subunits, VirA, VirFG and VirH (the published cluster [14] is incomplete, as modules for starter unit selection and introduction of proline have not yet been identified). The system incorporates many features characteristic of this second class of modular PKS, including a *trans*-acting acyl transferase VirI, duplicated domains (ACPs of modules 1 and 5 and peptidyl carrier proteins (PCPs) of module 8), nonribosomal peptide synthetase (NRPS) modules (3 and 8), an inactive domain (KS^o^ of module 9), and a set of *trans*-acting enzymes which introduce a β-methylation into the chain.

Nature has, in fact, evolved two distinct types of modular PKSs, referred to as *cis*-AT (including the erythromycin PKS (Figure 3)) and *trans*-AT (Figure 4). The principle distinguishing feature for *trans*-AT systems is the absence of an AT domain integrated into the subunits, as the activity is instead present as a discrete protein which acts iteratively to furnish extender unit to the modules [11]. Other characteristic features include unusual domain orderings, duplicated and inactive domains, atypical enzymatic functions, and modules distributed between two subunits (so-called ‘split modules’). This architectural divergence in all likelihood reflects independent evolutionary paths of the two types of systems [12], although more recent evidence indicates that some *trans*-AT PKSs may have evolved from a *cis*-AT parent [13].

In terms of stereochemical considerations, however, they are largely the same for the two systems, as stereochemistry can be introduced at several points within the pathways. For example, although fatty acids are constructed primarily from malonyl-CoA units, the AT domains of *cis*-AT PKSs exhibit specificity towards a number of branched extender units (including methylmalonyl-CoA, ethylmalonyl-CoA, hydroxymalonyl-ACP, methoxymalonyl-ACP, etc. [15–16]), thus incorporating pendant functionality into the polyketide skeleton (C-2-methyl, C-2-ethyl, C-2-hydroxy and C-2-methoxy groups, respectively). In the case of erythromycin A (**1**) (Figure 2 and Figure 3), for example, the C-2-methyl groups resulting from use of methylmalonyl-CoA exhibit both possible stereochemistries. In contrast, the majority of *trans*-ATs operating in *trans*-AT PKSs are specific for malonyl-CoA, although exceptions do exist (such as the ethylmalonyl-CoA-specific AT from kirromycin biosynthesis) [17]; C-2-methyl groups in these systems are thus introduced primarily by methyl transferase domains [18], with presumably defined the stereospecificity (the stereochemistry is not always evident, as it can be obscured by subsequent dehydration). The suite of processing reactions also introduces stereochemistry into the molecules: the hydroxy groups resulting from ketoreduction of the initially-formed C-3-ketones exhibit both configurations, dehydration of the hydroxy functionality generates both *cis*- and *trans*-double bonds, and finally, enoyl reduction can produce both configurations at the saturated C2-methyl centers. Other types of processing reactions present in *trans*-AT PKSs and certain *cis*-AT PKSs (for example, pyran synthase domains [19–20], double bond shifting modules [21–22], C-2-hydroxylases [11], etc.) can also have stereochemical consequences, but these will not be treated here as little is known to date about the enzymatic factors controlling the configurational outcomes. Finally, where chirality is introduced, post-PKS processing reactions also proceed with defined stereochemistry, although this aspect will also not be discussed in this article. The following sections will address the role of each of the principal PKS domains in controlling these stereochemical features, highlighting in each case the contribution of chemical biology in illuminating enzymatic function.

### Acyl transferases

Pathways to both the (2*R*)- and (2*S*)-isomers of methymalonyl-CoA exist in bacterial cells, and so in principle, the observed methyl configurations in the final polyketide products could arise by judicious choice by the PKS AT domains of one or the other enantiomer. The first information on extender unit selection in polyketide biosynthesis was provided in the mid-1980s via feeding of isotopically-labeled precursors to whole cells of the erythromycin producer *Saccharopolyspora erythraea*, leading to the generation of isotopically-labeled (2*R*)- and (2*S*)-methylmalonyl-CoA in situ [23]. When a precursor of (2*S*)-methylmalonyl-CoA, [2-^2^H_2_,2-^13^C]propionate, was used, analysis of the products by difference ^13^C{^1^H,^2^H} NMR provided evidence for isotopic labeling at C-2, C-4, and C-10 of the macrolide ring. This result was consistent with incorporation of (2*S*)-methylmalonyl-CoA during the second, fifth, and sixth chain extension cycles, with inversion of configuration at the C-2 center as found for fatty acid biosynthesis (vide infra) [24]. However, attempts to illuminate the origin of the remaining centers by feeding of ethyl [2-^2^H_2_,2-^13^C]-succinate to produce labeled (2*R*)-methylmalonyl-CoA in situ, were inconclusive.

**Figure 5:**
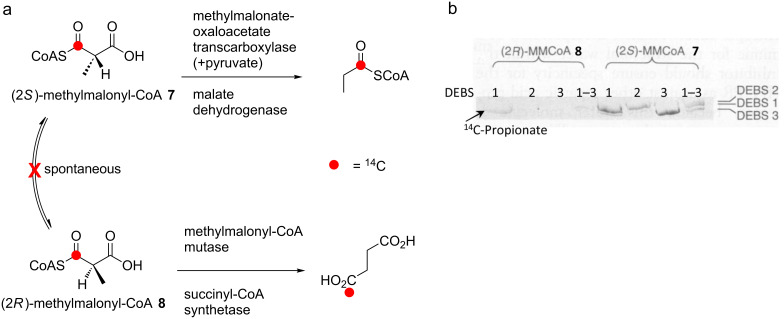
Determination of the stereochemistry of extender unit selection by the AT domains of modular PKS. a) Enantiomerically pure ^14^C-labeled (2*S*)-methylmalonyl-CoA (**7**) and (2*R*)-methylmalonyl-CoA (**8**) were generated enzymatically from a racemic mixture by consumption of the opposite enantiomer under conditions designed to minimize spontaneous epimerization. b) Results of labeling of the DEBS proteins with pure ^14^C-(2*R*)- or (2*S*)-methylmalonyl-CoA. Only incubation with (2*S*)-methylmalonyl-CoA (**7**) produced radioactive labeling of the three DEBS proteins (the signal obtained for DEBS 1 with (2*R*)-methylmalonyl-CoA (**8**) is due to the presence of a small amount of propionyl-CoA contaminant, which labeled the loading module. Image adapted from [26].

Access to the erythromycin PKS (DEBS) multienzymes as pure proteins [25] allowed extender unit preference in *cis*-AT PKSs to be investigated under more controlled conditions in vitro. Critically, the researchers were able to generate exclusively ^14^C-(2*S*)- or (2*R*)-methylmalonyl-CoA (**7** and **8**, respectively) by enzymatically removing the enantiomeric substrate under conditions designed to minimize spontaneous epimerization. Using the resulting enantiomeric materials, it was then shown by autoradiography that acylation of all six DEBS proteins is highly specific for the (2*S*)-isomer (**7**) [26], implying that the six AT domains present in the multienzymes select exclusively this stereoisomer (Figure 5).

**Figure 6:**
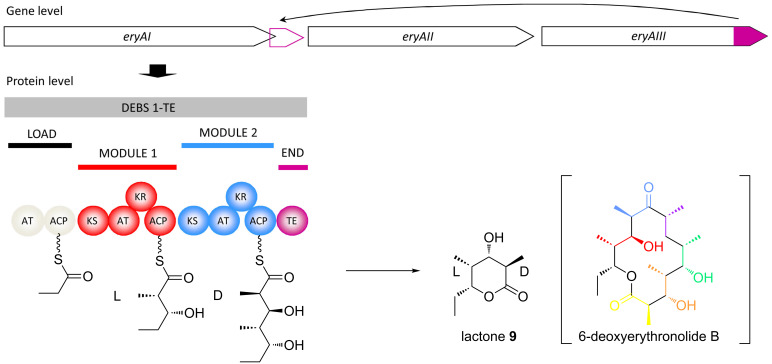
Creation by genetic engineering of the DEBS 1-TE model system. The region of the *eryAIII* gene encoding the thioesterase (pink) was relocated to the end of gene *eryAI*. The resulting protein, DEBS 1-TE, produces a small triketide lactone **9** instead of the heptaketide 6-deoxyerythronolide B. The two methyl centers in lactone **9** are of opposite stereochemical configuration, and thus DEBS 1-TE is an attractive protein for studying the control of stereochemistry.

Subsequent studies in vitro with a model recombinant protein, DEBS 1-TE (Figure 6), confirmed that this preference is also exercised during chain extension [27]. The DEBS 1-TE protein was created by joining the terminal TE domain to the end of the bimodular first subunit, DEBS 1 [28], to cause release of the polyketide at the triketide stage. This modification results in an experimentally tractable δ-lactone **9** instead of the 14-membered macrolide, 6-deoxyerythronolide B. Notably, the two methyl centers in the lactone have opposite configurations (for clarity, that at C-2 will be referred to as D-configured, and that at C-4 as L), and thus DEBS 1-TE represented an ideal system for elucidating the origin of the two configurations. In the presence of a suitable starter unit such as propionyl-CoA, (2*R*)-methylmalonyl-CoA as extender unit and NADPH (the cofactor for the KR domains), no product was observed. However, when (2*S*)-methylmalonyl-CoA was provided instead, the product was obtained at a satisfactory rate, showing that both modules select this isomer. Thus, the idea that one of the methyl configurations arises from use of (2*R*)-methylmalonyl-CoA by the analyzed modules and the second from use of the (2*S*)-isomer, was now firmly excluded. Given the high level of homology among many AT domains from *cis*-AT PKSs [29], it is likely that all such acyl transferases exhibit the same stereospecificity.

**Figure 7:**
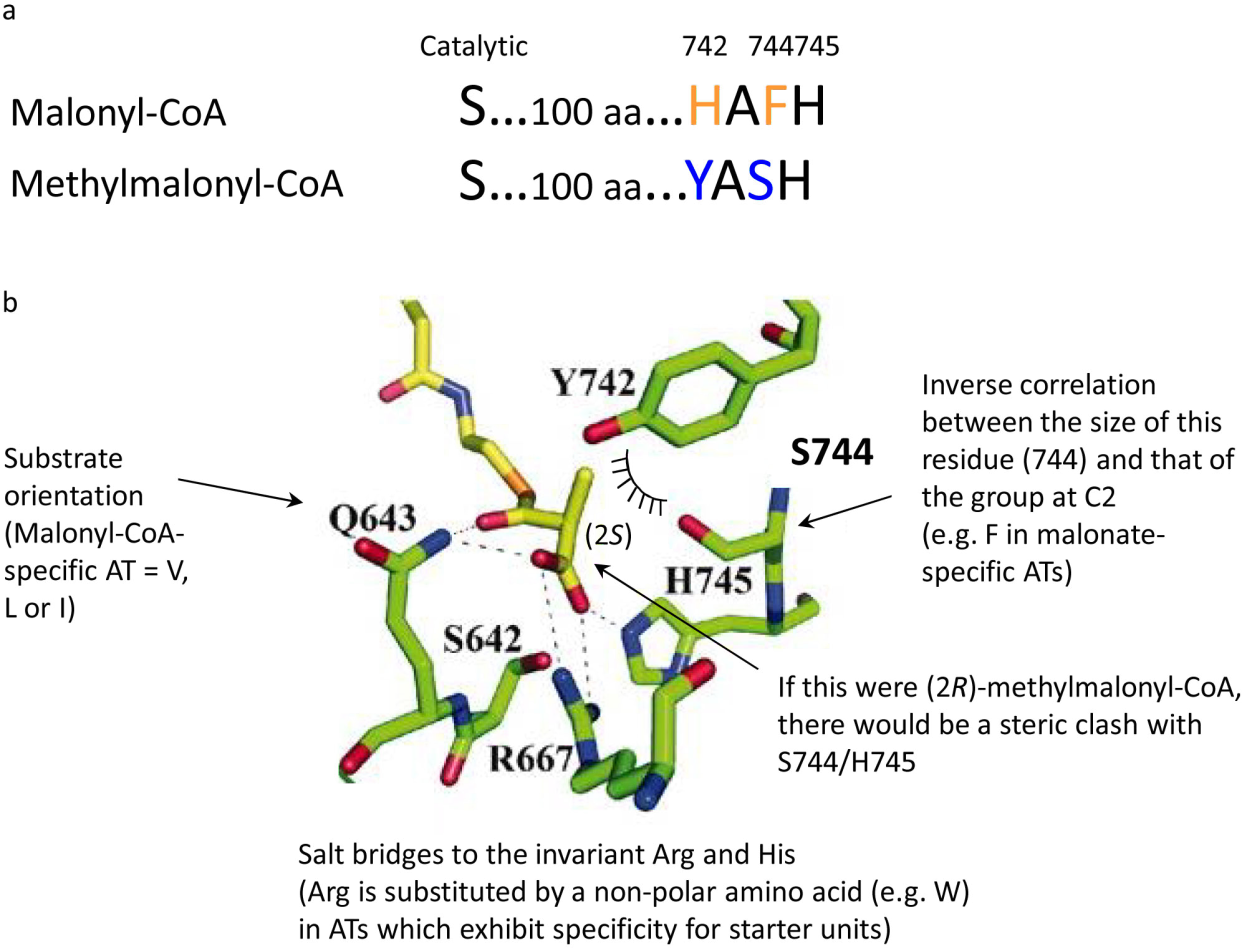
Model for substrate selection by AT domains. a) Sequence motifs in malonyl- and methylmalonyl-CoA-specific ATs which correlate with substrate choice. A HAFH motif is present some 100 amino acids downstream of the active site serine in malonyl-CoA-specific AT domains, while the corresponding sequence is YASH in methylmalonyl-CoA-specific ATs. The numbering is as in DEBS 3 (domain AT_5_). b) Model for the molecular basis of specificity for (2*S*)-methylmalonyl-CoA based on the crystal structure of DEBS AT_5_ solved in the presence of acetate [38]. This shows notably the proposed role of the Y, S and H residues of the conserved recognition motif. Reprinted with permission from [38]. Copyright 2006, National Academy of Sciences.

The AT domains operate by a ping-pong bi-bi mechanism [30], in which the initially formed acyl-*O*-AT intermediate is subject to nucleophilic attack by the terminal phosphopantetheine thiol of the ACP domain. Recent steady-state kinetic analysis of an AT domain sourced from DEBS module 3 (Figure 3) has provided evidence that the specificity for (2*S*)-methylmalonyl-CoA is expressed during the first half reaction of the ping-pong mechanism (i.e., formation of the methylmalonyl-*O*-AT intermediate) [30]. Substrate preference can be rationalized, at least in part, by bioinformatics which has revealed several sequence motifs correlating with building block choice (whether for starter or extender units, malonyl or branching extender units) [31–37], in combination with structure elucidation at high resolution of AT_5_ from the DEBS PKS, which was solved in the presence of acetate (Figure 7) [38].

For example, extender unit-specific ATs contain positively charged residues in the active site (R667 and H745, DEBS AT_5_ numbering) capable of forming salt bridges with the carboxyl group of the building block, while these are non-polar amino acids in starter-unit specific ATs. The choice of methylmalonyl-CoA over malonyl-CoA is correlated with a YASH motif some 100 residues downstream of the active site serine, whereas malonyl-CoA specific ATs exhibit an alternative HAFH sequence (Figure 7a) [38]. In the AT_5_ crystal structure, the Tyr, Ser and His all lie within the active site (the His is the second member of the catalytic dyad). This leads to a model in which the C-2-methyl of methylmalonyl-CoA forms favorable hydrophobic interactions with the Tyr while being sterically accommodated by the relatively small Ser (Figure 7b). Finally, stereospecificity for the (2*S*)-isomer appears to lie in steric clashes that would occur between a (2*R*)-methyl group and both the Ser and His of the YASH motif. Nonetheless, efforts in vivo to convert methylmalonyl-CoA-specific ATs into malonyl-CoA-specific ATs by exchange of these key sequence motifs resulted only in promiscuous ATs capable of recognizing both extender units [32,34,36], revealing that further elements of the AT active site contribute to specificity.

In terms of the stereochemistry of the less common extender units, labeling studies indicate that the (2*S*) isomer of ethylmalonyl-CoA is also used [39], which correlates with it originating predominantly from the reductive carboxylation of crotonyl-CoA [40]. Several extender units including aminomalonyl-ACP [41] and hydroxy-/methoxymalonyl-ACP [42] are generated via multi-step pathways from a primary metabolite, with the intermediates tethered to a discrete ACP domain. The building blocks are then transferred onto the AT domains of the PKS, and from there to the downstream integral ACP to participate in chain extension. Based on the presumed biosynthetic origin of these extender units (from L-serine and from a glycolytic intermediate (in all likelihood 1,3-biphospho-D-glycerate), respectively), it was initially proposed that the (2*S*)-isomer of aminomalonyl-ACP and the (2*R*)-isomers of hydroxyl-/methoxymalonyl-ACP [39] are employed. However, more recent crystallographic work on the zwittermicin pathway [43] in which hydroxymalonyl-ACP is used as extender unit, has raised some uncertainty over the hydroxymalonyl stereochemistry, as the (2*S*)-isomer would appear to fit better within the investigated AT structure. Indeed, selection of the (2*S*)-isomer, and correspondingly, the (2*R*) isomer of aminomalonyl-ACP, would simplify the biosynthetic mechanism in a number of polyketide pathways, as subsequent epimerization of the resulting pendant centers (vide infra) would not be required.

### Ketosynthases

**Figure 8:**
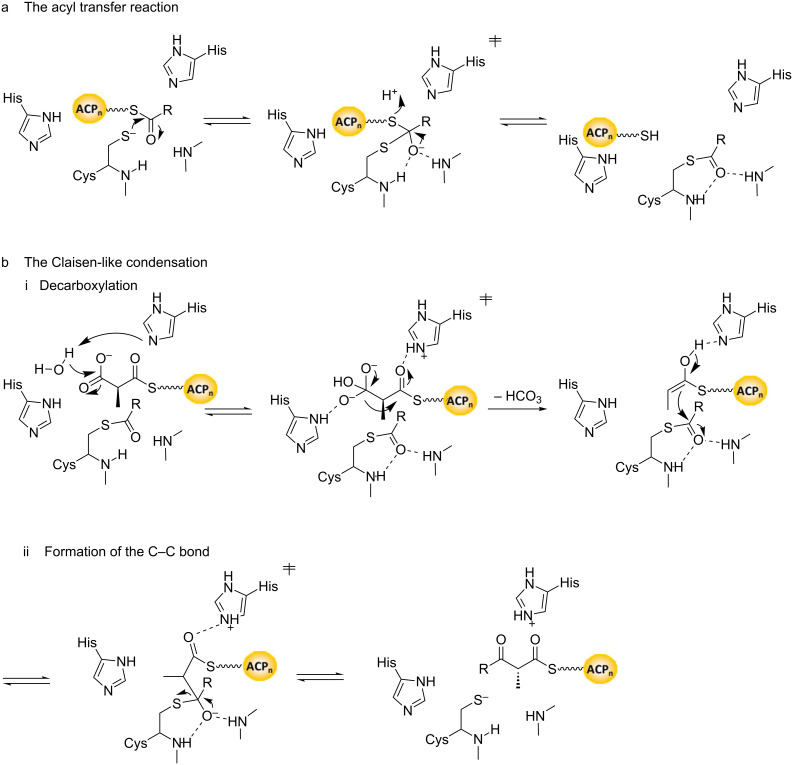
Proposed mechanism for KS-catalyzed chain extension, based on extrapolation from studies on homologous enzymes from animal FAS [44]. The reaction encompasses two stages overall: a) acyl transfer, and b) the Claisen-like condensation. From the stereochemical perspective, the important aspect of the mechanism is that the C-2-methyl stereochemistry is set by the direction of attack of the enolate nucleophile on the acyl enzyme carbonyl (reaction bii). (Although several elements of this mechanism differ from that proposed more recently in [45], including the roles of the His residues in the acyl transfer reaction, and whether decarboxylation proceeds with initial attack by a water molecule, these do not have stereochemical consequences).

The next step in the biosynthetic cycle is KS-catalyzed chain extension. This reaction occurs by nucleophilic attack of an enolate generated by decarboxylation of an ACP-bound extender unit onto the starter unit or chain extension intermediate attached to the active site cysteine of the KS domain. The face of the enolate which is used for the attack determines whether the reaction occurs with retention or inversion of configuration at the C-2 center relative to the starting material (Figure 8).

**Figure 9:**
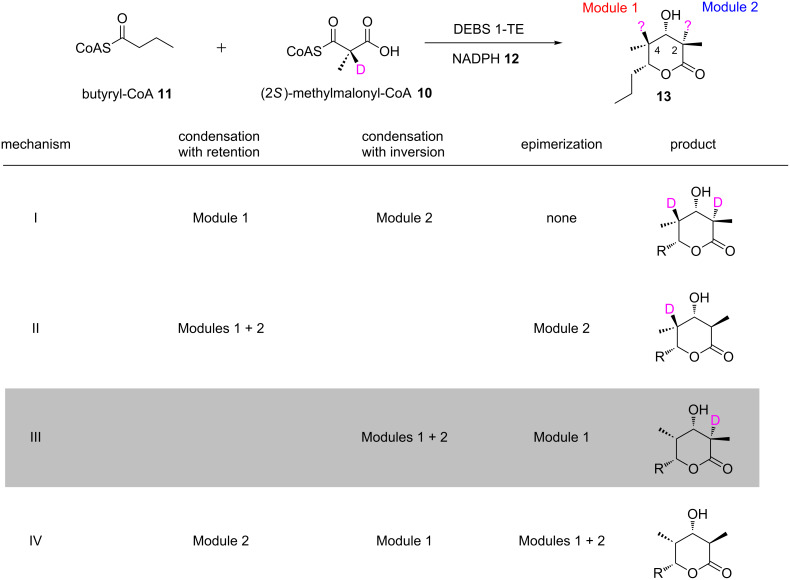
Experiment in vitro to determine the stereochemistry of condensation in modular PKS [46]. Use of specifically C-2-deuterium labeled extender unit **10** during biosynthesis with DEBS 1-TE (alongside starter unit butyryl-CoA **11** and NADPH **12**), resulted in a labeling pattern in the triketide lactone product **13**, which allowed discrimination between the four possible mechanisms for condensation in modules 1 and 2 of the PKS (the C-2 methyl center of the product is established by module 2 and the C-4 center by module 1). The obtained pattern (exclusive deuterium labeling at the C-2 position) was consistent with mechanism III (boxed) – inversion of stereochemistry in both modules as found for fatty acid synthase, with an additional epimerization occurring in module 1 to give the observed final configuration.

In the related FAS enzymes, this reaction has been shown to proceed with inversion of stereochemistry at the extender unit C-2 [24]. Circumstantial evidence for this same condensation stereochemistry in *cis*-AT PKSs was obtained for at least a subset of modules in the DEBS PKS by the feeding studies in *Sac. erythraea* cited previously, but direct proof that inversion occurs was provided by experiments in vitro with DEBS 1-TE [46]. In this study (Figure 9), (2*RS*)-[2-^2^H]methylmalonyl-CoA (**10**) was prepared and provided to DEBS 1-TE (along with starter unit butyryl-CoA (**11**) and NADPH (**12**)), knowing that solely the (2*S*)- isomer would be utilized. Analysis by mass spectrometry and NMR of the triketide lactone product **13** showed that only a single deuterium label was retained at the C-2 position bearing the D-configured methyl group (generated by module 2), while no labeling was observed at C-4 bearing the L-configured methyl group (generated by module 1). The opposite labeling pattern was obtained when biosynthesis was carried out with unlabeled (2*RS*)-methylmalonyl-CoA in D_2_O.

These labeling patterns are consistent with inversion of stereochemistry occurring in both modules 1 and 2 as in fatty acid biosynthesis without cleavage of the C-2–H bond (giving directly the D-configuration at C-2 observed in the final product), but show that an additional epimerization step must occur in module 1 to yield the L-methyl stereochemistry present at C-4 (thus explaining the loss of deuterium from the 2-position when (2*RS*)-[2-^2^H]methylmalonyl-CoA was used, and its incorporation from solvent in the presence of unlabeled extender) (mechanism III, Figure 9).

**Figure 10:**
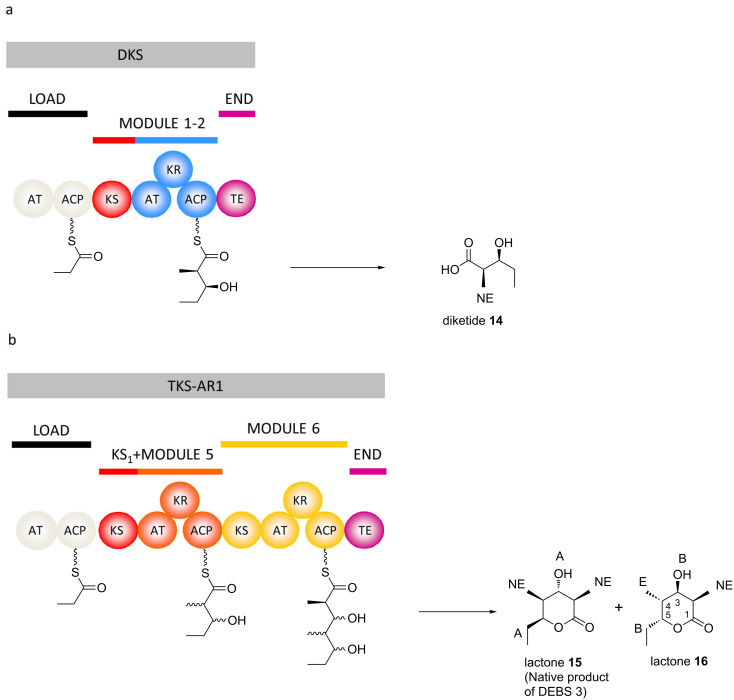
Genetic engineering experiments which suggested a role for the KS domain in epimerization. a) A diketide synthase (DKS) was created by attaching the loading module and KS_1_ of DEBS to the remainder of DEBS module 2, which was itself fused to the thioesterase (TE) domain [47]. The resulting construct yielded diketide **14** in which the methyl group at C-2 was not epimerized (NE). As the diketide generated by module 1 normally incorporates an epimerized methyl, this result was taken as evidence that KS_1_ can produce both epimerized and unepimerized methyl groups, and that the downstream KR ‘chooses’ which one is taken on as a substrate for reduction. b) In construct TKS-AR1, the same DEBS loading module-KS_1_ region was used to replace the initial KS of DEBS 3 [48]. The resulting protein produced two lactones: lactone **15**, the native product of DEBS 3 in which no methyl epimerization has occurred (NE) and the two hydroxy groups are A-type, and lactone **16**, in which the stereochemistry at the C-4 methyl center generated by module 5 is inverted (E). The presence of this epimerized methyl causes the direction of reduction to reverse (to B-type) in both modules 5 and 6, even though the methyl center produced by module 6 (C-2) is of native, non-epimerized stereochemistry (NE).

Although these experiments established the stereochemistry of condensation, showing it to furnish directly the D-methyl groups of polyketides, the origin of the epimerization activity in module 1 remained obscure. Shortly thereafter, the results of genetic engineering experiments carried out on DEBS KS_1_ implicated this domain as the seat of this activity, with a downstream KR then choosing between the two methyl configurations presented to it by the KS. More specifically, when KS_1_ was paired with the remaining domains of DEBS module 2 (AT_2_, KR_2_ and ACP_2_), and the hybrid module sandwiched between the DEBS loading module and the TE, the resulting construct produced a diketide **14** with opposite stereochemistry to that normally generated by module 1 (Figure 10a) [47]. This result was taken to show that KS_1_ can produce both methyl stereochemistries, but that in the hybrid 1/2 diketide synthase, the selectivity of KR_2_ for the *un*epimerized methyl configuration masks the KS_1_ epimerase activity. Subsequent work seemed to strengthen the idea that KS_1_ acts as an epimerase [48]. In this case (Figure 10b), the loading module-KS_1_ portion of DEBS 1 was grafted onto DEBS 3 (whose two modules 5 and 6 generate the unepimerized methyl configuration) to generate a hybrid PKS called TKS-AR1, and the stereochemistry of the resulting triketide lactones **15** and **16** established by NMR. This analysis showed that the methyl group arising from the hybrid 1/5 module was epimerized in 50% of the product **16** and that this change in stereochemistry was propagated to the ketoreduction in module 6, despite the lack of methyl group epimerization in this module. Thus, it appeared from these experiments that introduction of KS_1_ into a normally non-epimerizing context was sufficient to alter the methyl configuration, consistent with its role as an epimerase. However, as it has now been clearly established that it is instead the KR domains that possess this activity, it must be assumed that the engineered synthase suffered a significant change in architecture which allowed the epimerization to happen spontaneously, perhaps by providing water with increased access to the chain extension intermediates. How the KRs were shown to participate in epimerization will be detailed below.

### Ketoreductases

**Figure 11:**
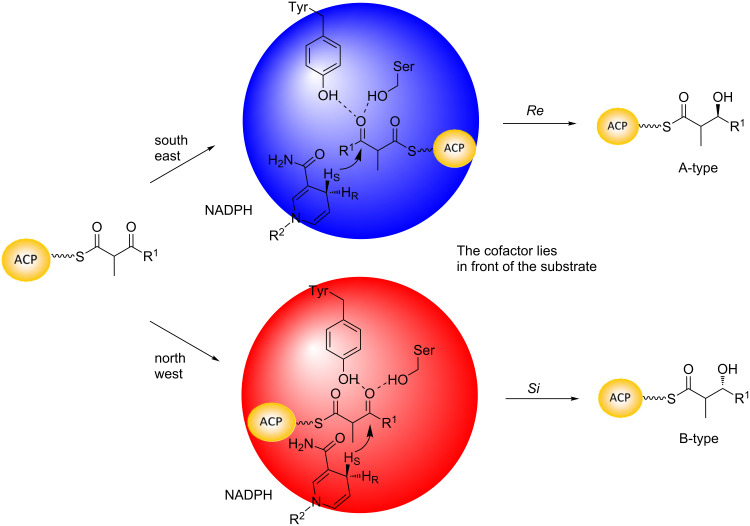
Models for control of the stereochemistry of reduction by KR domains. The two directions of ketoreduction achieved by a conserved catalytic apparatus (i.e. positions of the catalytic tetrad and NADPH cofactor) are obtained by entry of the ACP-bound substrate from one or the other side of the active site [68]. ‘South east’ entry gives reduction on the *re* face and an ‘A-type’ product, while the alternative ‘north-west’ entry yields *si* face reduction, and a ‘B-type’ hydroxy product.

KR domains catalyze the stereospecific reduction of the C-3-ketone groups arising from the chain extension reaction, to give both possible stereoisomers of the resulting hydroxy groups. The direction of reduction is intrinsic to the KR domains, as the majority of KRs transplanted by genetic engineering into alternative contexts have maintained their native stereospecificity [49–51]. Incubation of enzymatically-generated, chirally deuterated NADPH (both (4*R*)- and (4*S*)-[4-^2^H]NADPH) with modules 1, 2, 5 and 6 from the DEBS PKS and analysis of the resulting products by GC-MS, showed that all of the KRs are specific for the 4′-*pro*-*S* hydride of the nicotinamide cofactor [52–53], as found for fatty acid biosynthesis [54–55]. Given the high sequence similarity among KRs from modular PKS systems, it is likely that this hydride specificity is common to all of them. Indeed, the 8 KR structures solved to date (7 from *cis*-AT PKSs [56–62] and 1 from a *trans*-AT PKS [63]) show the domains to adopt the same overall fold and share a conserved active site architecture. These analyses have revealed the KRs to be monomeric proteins containing a catalytic subdomain and a catalytically-inactive structural subdomain, both of which exhibit a Rossmann fold. Within the catalytic subdomain, all reductase active KRs possess the active site tetrad of Tyr, Ser, Lys and Asn [64] characteristic of the short-chain dehydrogenase/reductase (SDR) superfamily [65–66], and bind the NADPH cofactor in the same orientation so that it presents its 4′-*pro*-*S* hydride to the active site. Therefore, the alternative directions of ketoreduction (referred to as A- and B-type [67] to avoid ambiguity, as the *R*/*S* designations can vary depending on the relative priority of the functional groups) are thought to arise from opposite modes of binding into the common active center (i.e., the binding modes are related by a 180° rotation around the axis of the target carbonyl, with either the *re* or *si* face of the C-3-keto group presented to NADPH) (Figure 11).

Achieving these alternative modes of binding necessitates that the substrate enter from one or the other side of the KR, as appropriate. Several sequence motifs (referred to here as the ‘Caffrey motifs’) correlating with the direction of reduction and therefore presumably guiding substrate entry, were initially identified by comparative sequence analysis [64,67], and shown subsequently by structural analysis to occupy positions proximal to the active site [56–62]. The strongest indicator for a B-type KR domain is an LDD motif in the region between amino acids 88 and 103 (numbered as in [67]) which is absent from A-type KR domains (B-type KR domains in *trans*-AT PKSs appear only to conserve the second D [63]). These residues lie on a flexible loop (the ‘lid loop’) adjacent to the active site. Additional amino acids in the 134–149 region, specifically P144 and N148, correlate with B-type KRs, while W141, which is located on the opposite side of the substrate-binding groove to the LDD motif, is most strongly indicative of an A-type KR. Nonetheless, despite the availability of multiple ketoreductase structures, the role of these residues in shepherding the substrates into their correct orientations remains unclear, possibly because none of the KRs was co-crystallized as a ternary complex with both native polyketide intermediate and cofactor.

To date, two alternative mechanisms have been proposed to account for substrate positioning. In the first [57], ‘southeast’ entry (A-type reduction) is the default, and from this direction the phosphopantetheine arm of the ACP can contact the conserved W. In B-type KRs, on the other hand, the southeast side of the active site is blocked by an interaction between the LDD and the ‘lid helix’ (a mobile α-helix adjacent to the NADPH cofactor), which prevents the phosphopantetheine arm from slipping between them. The intermediate therefore enters the active site from the ‘northwest’ side, where the phosphopantetheine can make favorable interactions with the conserved Leu. In the alternative proposal [62], the direction of reduction is controlled by a divergent degree of ordering within the active sites of A- and B-type domains. In A-type KRs, cofactor binding generates a well-organized and catalysis-ready active site, in which a key residue (Met in the solved structure upon which the mechanism was based [62]) blocks entry from the northwest, allowing the substrate to penetrate the active site groove only from the southeast. The characteristic W of this type of KR points into the southeast entry channel, where it may help orient the phosphopantetheine cofactor by hydrogen bonding. In contrast, in B-type KRs, cofactor binding is loose, allowing in principle the polyketide to enter from both sides of the channel. However, only binding of substrate from the northwest side results in a catalysis-competent conformation of the active site. In this model, the LDD motif does not interact directly with substrate, but may contribute to substrate-assisted assembly of the active site. (For more recent ideas on substrate guiding, see [69]).

**Figure 12:**
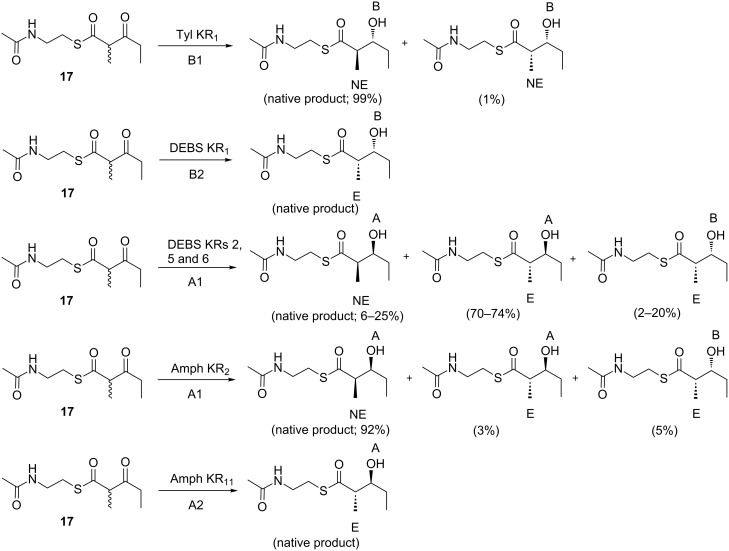
Assays in vitro to evaluate the stereospecificity of recombinant KR domains. A series of KR domains of all types (A1, A2, B1 and B2, as indicated) were investigated using a common substrate, racemic diketide derivatized as its *N*-acetylcysteamine (SNAC) thioester **17**. When the KR domains selected the appropriate substrate (C-2 non-epimerized (NE) for the type 1 domains and C-2 epimerized (E) for the type 2 domains), reduction occurred almost exclusively in the correct direction. However, when substrate bearing the non-native C-2 stereochemistry was chosen, the opposite direction of reduction was also observed. The percentages of each product obtained are shown.

Although KRs catalyze reduction from one or the other direction in their native contexts, for many KRs, this strict control is at least partially lost in vitro. Assays of KR activity have been carried out with model synthetic substrates in the context of native and engineered modules [48,70–71] and with KRs obtained as isolated domains [58,61–63,68,72–76]. In the majority of cases, the substrate used was the synthetically accessible (2*RS*)-2-methyl-3-oxopentanoic acid *N*-acetylcysteamine (NAC) thioester (‘β-keto diketide’) **17** – a racemic analogue of the diketide generated by condensation of a propionyl starter unit and a (2*S*)-methylmalonyl extender unit. NAC was chosen as the activating group because it mimics the terminal portion of the phosphopantetheine cofactor to which the chain extension intermediates are normally tethered. The stereochemistry of the reduction products was typically established by GC–MS and comparison to authentic synthetic standards, or alternatively by LC–MS. Analysis of results obtained with KRs from the DEBS [68], tylosin (Tyl) [68,72] and amphotericin [58,61] PKSs (Figure 12), showed that when the KRs selected the correct stereoisomer at the C-2 methyl position, reduction occurred almost exclusively in the native direction; the same result was obtained for certain of these KRs with diketide and triketide intermediates generated enzymatically in situ on ACP domains [77], a process leading only to the correct C-2 methyl isomer (vide infra). However, when the incorrect methyl isomer was chosen and reduced (which in some cases was the kinetically favored outcome [68]), reduction occurred in both the native and reverse directions (Figure 12). Thus, in these instances, a change in methyl stereochemistry was sufficient to flip the substrate in the active site, suggesting that the energetic differences between the two binding modes are minor. (The caveat with these results is that reduction might still have followed the natural course even in the presence of the ‘wrong’ methyl stereochemistry if the substrates more closely resembled the native ones and/or the substrates were attached to an ACP domain (the result, for example, of tethering (2*RS*)-2-methyl-3-oxopentanoate to an ACP, has not been tested)). In any case, these data encouraged the view that mutation of a few key residues in the KR active sites might be used to alter reduction stereochemistry.

Site-directed mutagenesis can indeed modify the stereochemical outcome of ketoreduction, at least in vitro, showing that the altered residues do play some role in stereocontrol. This is notably the case for changes introduced into the two Caffrey motifs. For example, swapping the B-type motifs of DEBS KR_1_ for characteristic A-type residues, yielded a KR which catalyzed exclusively A-type reduction of β-keto diketide **17** [73]. Unexpectedly, however, carrying out the reverse changes with the A-type DEBS KR_2_ produced a mutant KR with increased wild type behavior towards the model substrate, and thus the targeted residues cannot be the sole determinants of the direction of ketoreduction. Similarly, high-throughput mutagenesis of the Caffrey motifs [74] and other residues identified as potentially participating in stereocontrol by sequence and/or structural analysis [58,61], failed to produce consistent results, with certain mutations leading to the predicted shift in stereochemistry, others again strengthening wild type behavior, and still others having no effect on the stereochemical outcome. Most importantly, introducing the same Caffrey motifs mutations into DEBS KRs 1 and 2 housed within DEBS 1-TE produced no discernable stereochemical switch in vivo [78]. These results clearly show that within the context of an intact PKS multienzyme, other factors override the effects of these mutations; possibilities include the specificity of one or more downstream domains acting against stereochemically altered intermediates, increased hydrolytic removal of stalled chains via the proof-reading activity of a cluster-associated TEII domain [79–81], or constraints imposed on substrate orientation due to the fact that the intermediates are tethered to ACP domains which are themselves covalently linked to the KRs; which if any of these mechanisms predominates remains to be determined. In the meantime, it has proven more successful to swap entire KR domains both within and between PKS multienzymes, as a means to achieve rationale alteration of C-3-hydroxy stereochemistry [49–51].

As noted previously, following chain extension in certain modules, the initial D-2-methyl group undergoes an epimerization reaction to yield the L-methyl. In mechanistic terms, epimerization involves removal of the C-2 proton and delivery of proton to C-2 from the opposite face of the resulting, planar enol/enolate intermediate. Monitoring by NMR of the rate of epimerization of a model C-3-ketoacyl ester (ethyl 2-methylacetoacetate) showed this reaction to be rapid at room temperature (*t*_1/2_ = 4.7 min) [82]. Thus, during polyketide biosynthesis, there must be some mechanism to protect the intermediate from spontaneous epimerization following chain extension, both as it is passed between the KS and KR active sites and within the KR prior to ketoreduction (the alternative possibility that epimerization occurs in all modules but that the KRs select the correct isomer is excluded by the in vitro studies with DEBS 1-TE (Figure 9) [46], as no deuterium from the deuterated extender unit would have been retained in the triketide lactone product). Sequestering of intermediates by the ACP domains has been proposed as a source of configurational stability at C-2 [82]. However, as all direct study of this question to date by NMR has failed to reveal any direct contact between modular PKS ACP domains and their attached substrates [83–85], the origin of this stabilization remains unknown.

**Figure 13:**
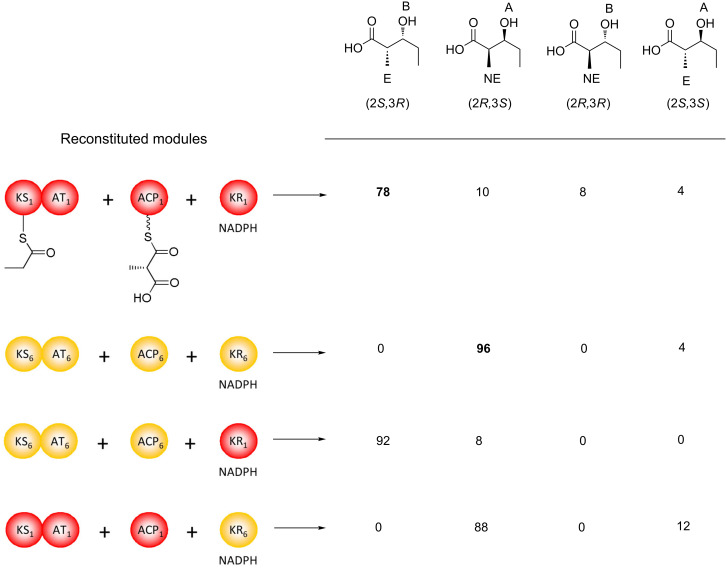
Assays in vitro which provided the first direct evidence that KR domains act as epimerases [77]. Biosynthesis in these experiments was carried out by reconstituted modules comprising KS-AT didomains and isolated KR and ACP domains. The KS was charged with the propionate starter unit from propionyl-SNAC, and the ACP with extender unit by the AT domain using (2*S*)-methylmalonyl-CoA. The product diketides were hydrolyzed from the ACP domains and their stereochemistries were determined by chiral GC–MS. Reconstituted DEBS modules 1 and 6 (red and yellow, respectively) gave predominantly the expected products (epimerized C-2 methyl and B-type alcohol stereochemistry for module 1; non-epimerized C-2 methyl and A-type alcohol stereochemistry for module 6 (indicated in bold)), while exchanging only the KR domain caused the reconstituted modules to produce the product characteristic of the introduced KR (for example (line 3), KR_1_ in place of KR_6_ resulted in the native product of module 1). Thus, the KRs were shown to control the stereochemistry at both the C-2 and C-3 positions of the chain extension intermediates.

KRs were first suggested to act as epimerases – despite the fact that no other SDR enzyme exhibits this activity – based on structural analysis [57]. This proposal led to the classification of PKS KRs working on C-2 methylated substrates into six distinct categories – KRs catalyzing A- and B-type reduction in the absence of epimerization (A1 and B1, respectively), KRs catalyzing both epimerization and the two senses of ketoreduction (A2 and B2), reductive- and epimerization-inactive KRs (C1), and KRs catalyzing epimerization in the absence of reduction (C2). (KRs operating on substrates lacking C-2 methyl groups are referred to as A0 and B0). The first direct proof for this activity was provided by studies on reconstituted modules (combinations of individually purified KS-AT didomains, ACPs and KRs) [77]. In brief, the notable finding of this work was that the stereochemical outcome both at C-2 and C-3 of the products correlated not with the modular origin of the KS-AT and ACP domains, but with that of the KR. For example, combining KS-AT and ACP from DEBS module 6 (which produces a non-epimerized methyl and an A-type C-3-hydroxy) with DEBS KR_1_ in the presence of starter unit (synthetic propionyl-SNAC), NADPH (**12**) and (2*RS*)-methylmalonyl-CoA, resulted in diketide with the stereochemistry at both the C-2 methyl group (epimerized) and C-3-hydroxy group (B-type) associated with module 1 (Figure 13). Conversely, mixing the KS-AT and ACP domains from DEBS module 1 (which produces an epimerized methyl and a B-type C-3 hydroxy) with the DEBS module 6 KR, yielded diketide incorporating the stereochemistry characteristic of module 6 (unepimerized C-2 methyl and A-type reduction at C-3) (Figure 13). Thus, these experiments provided the first conclusive evidence that certain KR domains can control the stereochemistry at both C-2 and C-3 of the chain extension intermediates.

**Figure 14:**
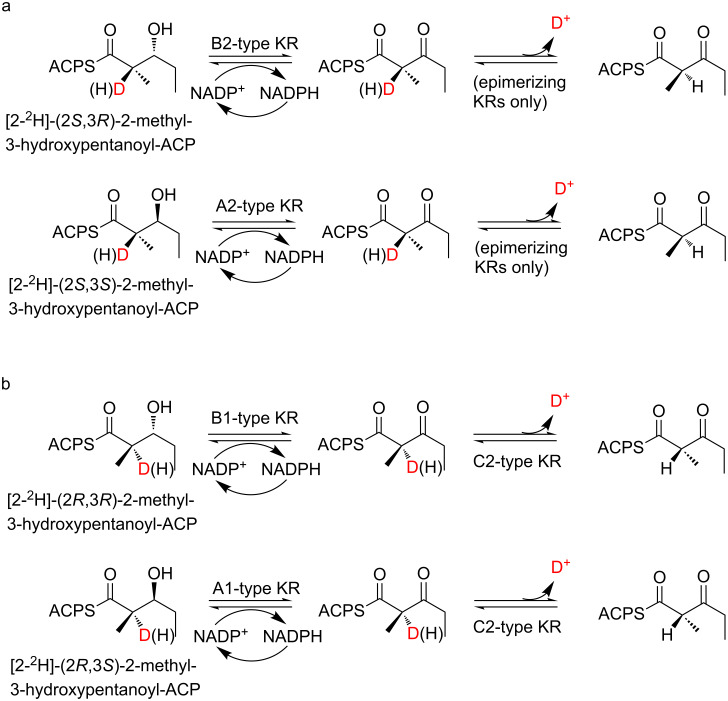
Assays in vitro to demonstrate directly the epimerase activity of PKS KR domains. a) Equilibrium exchange assay [86]. In these assays, an equilibrium is established between stereospecifically deuterated 3-hydroxy diketide-ACP (incorporating either (3*R*)- or (3*S*)-hydroxy stereochemistry as appropriate) and the 3-keto form, which then undergoes KR-catalyzed racemization at the C-2 center. This epimerizing activity is detected by LC–MS via time-dependent washout of deuterium from the reduced product. While A1 or B1-type KRs could catalyze the oxidation of the deuterated compounds, the deuterium would not be lost by subsequent epimerization. b) Tandem equilibrium exchange assay [87]. The aim of this assay is to demonstrate the intrinsic epimerization activity of non-reducing KRs (C2-type). As these are not capable of establishing the initial equilibrium between the C-3 hydroxy and keto forms of the substrate, an additional reducing but non-epimerizing KR (either A1- or B1-type) is added to the assays to carry out these step with (3*R*)- and (3*S*)-hydroxy substrates, respectively. The epimerizing capacity of the KR under study is then detected as for the classical equilibrium exchange assay described above.

The most convincing evidence has emerged from a series of so-called ‘equilibrium isotope exchange (EIX)’ experiments [86–88] – another clear illustration of the power of chemistry to elucidate key aspects of stereocontrol. In these assays (Figure 14a), the epimerization activity of select KRs (DEBS KR_1_, nystatin (Nys) KR_1_ and rifamycin (Rif) KR_7_) was demonstrated directly by incubating them with the stereochemically appropriate, configurationally stable reduced product obtained by chemical synthesis, in which C-2 was deuterium labeled (i.e., [2-^2^H]-2-methyl-3-hydroxypentanoate); the substrate was tethered enzymatically to a model ACP domain sourced from the DEBS PKS. By incubating with NADP^+^, the redox reaction was run in reverse, establishing an equilibrium between the oxidized (either (2*R*)- or (2*S*)-2-methyl-3-ketoacyl-ACP) and reduced forms. Under these conditions, time-dependent washout of deuterium from the C-2 position (above background) occurred for epimerizing KRs as they are capable of racemizing this position once the C-3-keto is present, while the label remained intact for two model, non-epimerizing KRs (DEBS KR_6_ and Tyl KR_1_), as confirmed by LC–MS analysis [89] of the reduced products (while chiral GC–MS was used to confirm that no change in configuration of the reduced product occurred).

This assay was subsequently extended to demonstrate the intrinsic epimerase activity of specific non-reducing KRs [87]. In this ‘tandem EIX’ format (Figure 14b), the ketoacyl substrate for the KR to be assayed is generated transiently from the appropriate reduced product by a second, validated non-epimerizing KR, at which point, the intrinsic epimerase activity of the target KR is again evidenced by time-dependent washout of the C-2 deuterium label. Using this coupled assay, epimerase activity was established for two natively non-reducing (C2-type) KRs (DEBS and pikromycin (PIKS) KRs 3), as well as redox-defective mutants of DEBS KR_1_ obtained by site-directed inactivation of the NADPH-binding site.

The tandem assay strategy was also used to try to identify residues potentially participating in the epimerization reaction [88]. This is an intriguing question, as comparative sequence analysis [57,90] fails to reveal any residues which are differentially and strictly conserved in epimerizing KRs relative to non-epimerizing KRs, which could serve as catalytic general acids and bases. In these in vitro experiments, it was possible to decouple the role of the active site Tyr and Ser residues in the reduction and epimerization reactions by assaying two natively redox-inactive but epimerizing KRs (DEBS and PIKS KRs 3; C2-type), along with a validated redox active but epimerization inactive KR domain (DEBS KR_6_). The fact that the mutant KRs lost a substantial percentage of their epimerase activity implicates both of these residues in the epimerization reaction. On the other hand, it is not clear how the same residues can function in both capacities – what, for example, inhibits the reduction occurring prior to epimerization if identical amino acids are involved? One possibility, which has not previously been discussed in the literature, is that in fact, epimerizing KRs bind their substrates in two distinct modes. In the first, which is only available to the substrate bearing the non-epimerized methyl center, the thioester and C-3-keto groups are aligned so that the p*K*_a_ of the C-2 proton is suitably depressed, allowing facile catalysis by KR residues or alternatively abstraction by an available water molecule. The resulting enol/enolate could tautomerize spontaneously back to the original substrate or its epimer, with only the epimerized substrate possessing the required C-2 methyl stereochemistry for subsequent reduction. This epimer would then bind in a second mode common to all KRs of the same type (i.e., either A- or B-), in which it is positioned properly relative to the reductive catalytic apparatus. In this way, the KRs could effectively discriminate between substrates bearing the two methyl stereochemistries. This mechanism might be borne out by the first crystal structures of epimerizing KRs in the presence of native substrate, and such data are eagerly anticipated. In the meantime, in the absence of a clear mechanistic basis for epimerization, it has been shown possible to rationally alter the methyl stereochemistry (both introducing and removing C-2 epimerization) by the whole-sale exchange of KR domains in the context of model PKS systems [51,61,91], although the efficiency of such experiments remains generally low.

### Dehydratases

**Figure 15:**
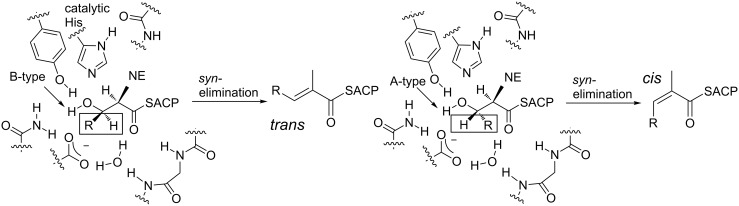
Model for DH-catalyzed generation of *trans* and *cis* double bonds by *syn* elimination from substrates bearing C-2-methyl groups (the side chains shown are those found in DEBS DH_4_ [92]). Both substrates incorporate a (2*R*)- (non-epimerized, NE) methyl group, but differ in the relative orientation of the C-3 H and R groups within the active site (boxed). In both cases, however, the hydroxy group is held in place by interactions with Tyr and Asp residues. *Syn* dehydration of the product derived from B-type reduction (indicated) directly yields a *trans* double bond, while *syn* dehydration of an A-type hydroxy substrate directly gives a *cis* double bond. Adapted from [95].

PKS DHs are members of the double hot dog (DHD) family of enzymes, in which the active site in one of the two fused single-hot dog subdomains is inactive [92–95]. The DHs catalyze the elimination of water from the polyketide intermediates to form double bonds which are typically *trans* (*E*) in configuration, although *cis* (*Z*) alkenes are also present in a significant fraction of structures [94]. Studies on the evolutionary related DHs from animal FAS which produce exclusively *trans* double bonds [96] have demonstrated that this reaction proceeds with overall *syn* elimination of the *pro*-(2*S*) hydrogen and the (3*R*)-hydroxy group [97–98], while biochemical and stereochemical experiments on this class of enzymes suggest a catalytic mechanism in which a single histidine plays the role of both general acid and base [97–99]. Extending this proposal to PKS DHs which operate on C-2 methylated intermediates, implies that only D-methylated ((2*R*), unepimerized) compounds will be substrates for the DHs, as then the C-2 proton is of the correct stereochemistry. In this model (Figure 15), whether *trans* or *cis* double bonds are obtained directly by *syn* elimination depends on the hydroxy configuration, with (3*R*)-hydroxy groups (B-type ketoreduction) leading directly to *trans* double bonds and (3*S*)-hydroxy groups (A-type) giving *cis* double bonds [95]. These alternative reaction courses could be achieved by a common mode of binding into the DH active site, where all that differs is the direction in which the remainder of the chain (R in Figure 15) points [95]. To summarize: According to this proposal, the KRs ultimately determine whether or not dehydration can occur (it should not occur for intermediates in which the C-2 methyl is epimerized, because the C-2 proton is inaccessible to the DH catalytic apparatus) and the stereochemistry of the resulting double bonds (via A- or B-type reduction on unepimerized chains; or in other words, (2*R*,3*R*) intermediates yield *trans* double bond stereochemistry, and (2*R*,3*S*), *cis* double bond stereochemistry).

A number of experiments reported to date support the origin of *trans* double bonds from a B-type hydroxy precursor. Specifically, studies in vitro with recombinant DH domains from the DEBS [100] and nanchangmycin [101] PKSs on ACP-tethered substrates generated in situ from reconstituted modules acting on synthetically-prepared diketide SNACs, showed that both domains generated *trans* double bonds from the corresponding (2*R*,3*R*)-2-methyl-3-hydroxyacyl chains. In the case of the DEBS DH, no reaction was observed for the three diastereomeric substrates (e.g., (2*S*,3*R*), (2*R*,3*S*) and (2*S*,3*S*)). A model DH from the Tyl PKS was also assayed with a panel of substrates, and found to recognize B-type and not A-type alcohols, generating exclusively *trans* double bonds [102].

**Figure 16:**
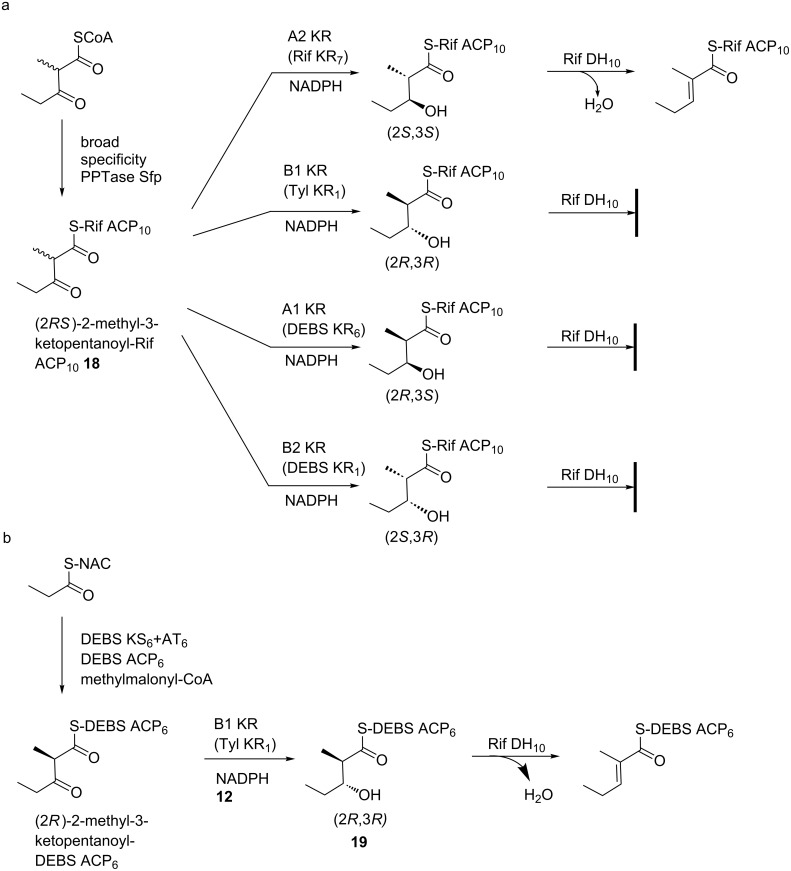
Stereospecificity of dehydration by Rif DH_10_ [94]. a) The four possible diastereomeric diketide-ACP substrates were prepared by stereospecific C-3 reduction (and C-2-methyl epimerization in the case of the A2 and B2-type KRs) of (2*RS*)-2-methyl-3-ketopentanoyl-Rif ACP_10_. Only the substrate bearing (2*S*,3*S*)-2-methyl-3-hydroxy stereochemistry was accepted as substrate by Rif DH_10_, yielding the *trans* double bond isomer. b) The alternative (2*R*,3*R*)-2-methyl-3-hydroxy substrate was prepared on DEBS ACP_6_ via condensation of propionyl and methylmalonyl building blocks by KS_6_, followed by stereospecific reduction by Tyl KR_1_. *Syn* dehydration again yielded the *trans* double bond.

On the other hand, the origin of *cis* double bonds is much less obvious. The only clear evidence to date for *cis* bond formation by a specific module comes from studies of the phoslactomycin PKS; here, only diketide incorporating a *cis* double bond was shown to productively prime the second chain-extension module, implying that it must be the product of the first module (which incorporates an A-type KR) [103]. *Cis* double bond formation was also explored by studies in vitro with a DH from module 10 of the Rif PKS [94]. In this case, the natural DH substrate had previously been shown to contain (2*S*,3*S*)-2-methyl-3-hydroxyacyl functionality [104], which is not in accord with the (2*R*,3*S*) stereochemistry postulated for *cis*-double bond precursors. Indeed, dehydration by recombinant Rif DH of this substrate (**18**) tethered to the native ACP domain resulted in a *trans* double bond, while none of the diastereomeric substrates were active (Figure 16a). Intriguingly, the diastereospecificity of the dehydration was completely reversed when the polyketide chain was attached to a non-cognate ACP from DEBS, with the (2*R*,3*R*) isomer (**19**) now being dehydrated to a *trans* double bond (Figure 16b). This is apparently the first example of such reversal of diastereospecificity due to the nature of the thioester conjugate. Taken together, these results agree with a common *syn* dehydration mechanism for PKS DHs, but the requirement that the abstracted proton be positioned equivalently relative to the conserved His for both the (2*S*,3*S*) and (2*R*,3*R*) polyketide chains, requires that the substrate enters from the opposite side of the DH active site. Nonetheless, this alternative direction of entry mechanism could explain the surprising observation that some A-type KRs are found in modules producing *trans* double bonds [105].

In any case, the obtained data failed to shed light on the origin of this *cis* double bond in the final rifamycin structure, although clearly it arises from isomerization of an initially formed *trans* alkene. Indeed, further studies on the Rif PKS and a handful of other systems have revealed that at least a subset of *cis* double bonds in the products arise from mechanisms other than direct DH-catalyzed dehydration. These include isomerization by either integral enoyl isomerase domains (present in *trans*-AT PKSs only) [21–22,106] or by post-PKS domains [107–109], and TE-mediated formation from a B-type alcohol precursor [110]. It may thus be the case that all PKS DHs produce *trans* double bonds. Consistent with this idea, comparative sequence analysis and the resolution of six DH crystal structures to date (1 from DEBS (*trans*-double bond producing module) [92], 4 from the curacin PKS (1 *cis* and 3 *trans*) [93], and 1 from the Rif PKS (*cis*) [94]) have not revealed any notable differences between DHs giving rise to *trans* double bonds and those apparently responsible for direct *cis* double bond formation. In terms of the catalytic mechanism, a two-base mechanism has been proposed based on the crystal structure of the DEBS DH [92], in which the conserved His acts as a general base to deprotonate at C-2, while an Asp residue serves as a general acid to stabilize the C-3 hydroxy leaving group. However, only the His has been shown by site-directed mutagenesis to be essential [111–112], and so definitive proof of whether the classic one-base mechanism mentioned earlier [97–99] or alternative two-base mechanism applies [113], remains to be obtained.

### Enoyl reductases

**Figure 17:**
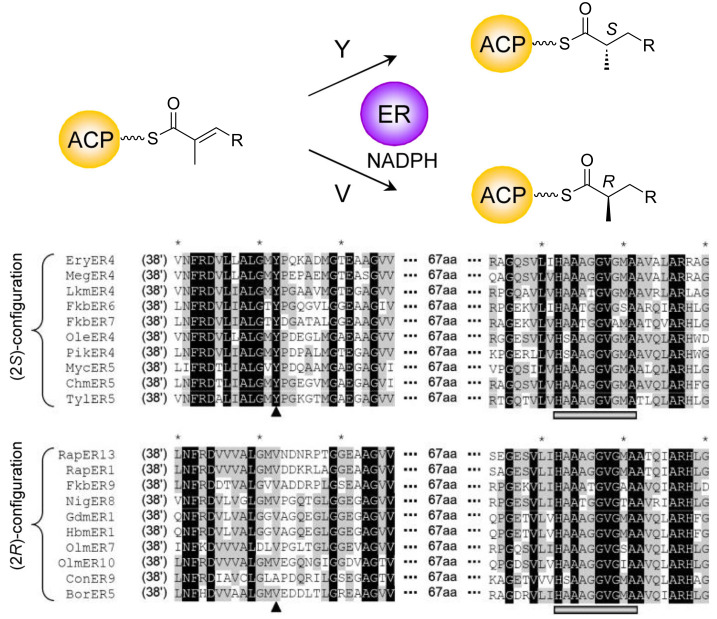
Stereocontrol by PKS ER domains. Sequence motifs correlated with the final stereochemistry of the C-2 methyl group [116]. When a conserved Y is present (indicated with the triangle), a (2*S*)-methyl stereochemistry is observed, while the presence of a conserved V at the same position correlates with (2*R*)-methyl stereochemistry. The grey bar indicates residues involved in binding the NADPH cofactor **12**. Residue numbering is based on that of *E. coli* QOR (PDB ID 1QOR). Reprinted and adapted with permission from [116]. Copyright (2010) American Chemical Society.

The enoyl reductase domains act on *trans* double bonds, producing fully-saturated methylene groups. In fatty acid biosynthesis by animal FAS, this reaction proceeds with attack of the 4′-*pro*-*R* hydride of NADPH on the 3-*re* face of the unsaturated thioester intermediate, with stereospecific protonation at the 2-*si* face, giving an overall *syn* addition [114]. In the case of polyketide chains, when a C-2 methyl substituent is present, enoyl reduction has stereochemical consequences, producing both the (2*R*)- and (2*S*)-configurations depending on which side of the double bond is protonated. As for the KR domains, by comparative sequence analysis of PKS ERs, a correlation was uncovered between the presence of specific residues and the direction of reduction [115] (Figure 17). When the identified position, which lies some 90 residues upstream of the conserved NADPH-binding motif, is occupied by a tyrosine residue, the methyl branch has an *S* configuration. In domains producing the alternative *R* configuration, this residue is most often valine, but also alanine or phenyalanine.

**Figure 18:**
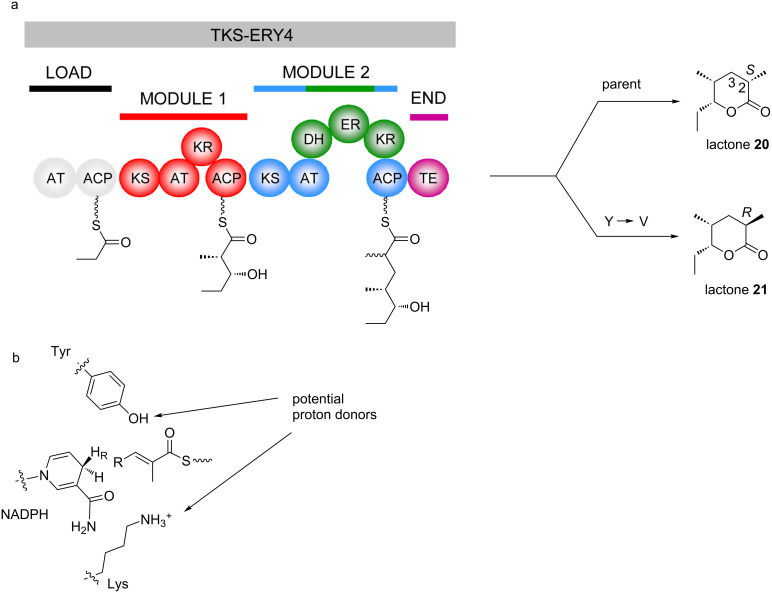
a) PKS engineered to test the role of the ER stereospecificity residues [115]. TKS-ERY4 was created by replacing the KR domain of DEBS module 2 (within the context of DEBS 1-TE) with the ‘reductive loop’ (DH-ER-KR) from DEBS module 4. This PKS gives rise to lactone **20** incorporating a (2*S*)-methyl group, consistent with the presence of the conserved Y in the active site. Site-directed mutation of this Y to the alternative V resulted in a complete shift in stereochemistry to give lactone **21**, with (2*R*)-methyl stereochemistry. b) Model for reduction by ER domains from the 4′-*pro*-*R* hydride of NADPH. When the Tyr is present, it acts as the proton donor following reduction to give the (2*S*)-methyl. In its absence, the Lys residue on the opposite side of the active site acts as the general acid, yielding the (2*R*)-methyl.

The role of these residues in stereocontrol was evaluated in vivo by site-directed mutagenesis of a derivative of DEBS 1-TE in which the KR domain of module 2 was replaced with the ‘reductive loops’ (DH-KR-ER tridomains) sourced from the DEBS and RAPS PKSs (giving TKS-ery4 and TKS-rap13, respectively) [115]. Native TKS-ery4 produces a triketide lactone **20** with a (2*S*)-methyl, while TKS-rap13 yields the alternative (2*R*)-methyl. Dramatically, when the conserved Y of the DEBS ER in TKS-ery4 was replaced with V, the resulting lactone **21** incorporated exclusively the (2*R*)-methyl (Figure 18a). This result showed that this residue is involved in ER stereocontrol. Nevertheless, the equivalent mutation introduced into the ER of TKS-rap13 (V to Y) did not result in the predicted change in stereochemistry at C-2 to *S*, with only parental product obtained. In subsequent experiments [116], 4 additional residues characteristic of (2S) specific domains were introduced simultaneously into the RAPS ER within the same model system, but this yielded only a small overall shift in stereochemical outcome.

On the other hand, mutagenesis of a putative catalytic residue (a Lys) without changing the Val had a more dramatic effect on stereochemistry [116]. To explain this result, it is proposed that the Lys serves as proton donor at C-2, and in its absence, there is less control of the face to which the proton is added from solvent to the carbanion intermediate. Based on the high-resolution structure of a representative ER from the spinosyn PKS [60], this mechanism has been extended to account for the role of the conserved Tyr (Figure 18b). In the solved structure, the Lys and the Tyr lie on opposite sides of the active site cleft from one another, in appropriate positions to protonate the C-2 carbon of a bound polyketide substrate. When the Tyr is present, it acts as the proton donor, but in its absence (as in the native PKSs in which V is instead present), the Lys delivers its proton from the opposite side of the polyketide substrate (thus explaining the reversal in stereochemistry observed with the Y to V mutant in TKS-ery4). Clearly, however, simply introducing Tyr at the appropriate position into (2*R*)-producing ERs (as in the experiments with TKS-rap13) is not sufficient to override proton donation by the Lys, and so rational manipulation of ER stereochemistry by site-directed mutagenesis awaits identification of further stereochemical determinants in ER active sites.

### Bioinformatics-guided structure elucidation

The strong correlations between certain sequence motifs present in PKS domains and the stereochemistry of the resulting polyketide chains has been exploited in several cases to predict and/or corroborate absolute stereochemical assignments made on newly-discovered natural products (for example, elansolid [117], the disciformycins [118], hygrobafilomycin [119], phormidiolide [120], and haprolid [121]), either by manual inspection of domain sequences or by more sophisticated methods including hidden Markov model (HMM)-based sequence classification (e.g., ScoreDiff [90]). This is notably the case for the direction of ketoreduction by assignment of KRs as either A- or B-type. Such analyses are relatively straightforward when the canonical ‘Caffrey’ motifs are present (for example, the LDD motif indicative of B-type KRs, with the second D being most diagnostic, particularly for *trans*-AT PKSs) and therefore these predictions can be an important complement to full structure elucidation. On the other hand, some KRs possess sequence features of both A- and B-type KRs, and so confident assignment is not possible [90,120]. In addition, predicting the configuration of the adjacent C-2-methyl groups (i.e., whether epimerization occurs or not) remains unreliable at present [90], but this situation may improve with the incorporation of additional sequences of epimerizing KR domains into sequence classification programs. In terms of predicting double bond stereochemistry, using the direction of ketoreduction as a guide is not an infallible method (as explained earlier, although A-type reduction is often correlated to *cis*-double bond formation and B-type reduction, to *trans*, the exact opposite outcome has been observed in multiple systems). This situation would be improved by elucidation of the complete set of molecular mechanisms underlying *cis*-double bond formation. For the ER domains, as described previously, the observed methyl configuration correlates quite strongly with the residue at a specific sequence position (Y = *S* configuration; not Y = *R*) [115], and this has already proved useful for correctly predicting methyl stereochemistry (see for example, [122–123]).

## Conclusion

As illustrated in this review, the tools of chemical biology coupled with molecular biological techniques have played a critical part in elucidating fundamental aspects of stereocontrol in modular polyketide biosynthesis. Given that our molecular understanding of these determinants remains incomplete – notably for the processing KR, DH and ER domains – this approach will undoubtedly continue to play an indispensable role in future work in this area.
